# Spectrum of inherited metabolic disorders diagnosed through newborn screening and symptomatic referrals in Eastern Türkiye

**DOI:** 10.1016/j.ymgmr.2026.101349

**Published:** 2026-09-04

**Authors:** Sezai Arslan, Filiz Ekinci Uğan, Oğuzhan Yaralı, Hasan Kahveci

**Affiliations:** a Department of Inherited Metabolic Diseases, Erzurum City Hospital, Erzurum, Turkey; b Department of Nutrition and Dietetics, Erzurum City Hospital, Erzurum, Turkey; c Department of Medical Genetics, Erzurum City Hospital, Erzurum, Turkey; d Department of Neonatology, University of Health Sciences Erzurum Medical Faculty, Erzurum, Turkey

**Keywords:** Inherited metabolic disorders, Newborn screening, Birth prevalence, Biotinidase deficiency, Phenylketonuria, Eastern Türkiye

## Abstract

**Objective:**

Inherited metabolic disorders (IMDs) are heterogeneous genetic diseases whose observed spectrum is influenced by population characteristics, newborn screening (NBS), and diagnostic pathways.This study aimed to characterize confirmed IMDs diagnosed through NBS and symptomatic referrals at a tertiary center in Eastern Türkiye and estimate the regional burden of selected disorders.

**Methods:**

This retrospective study included patients evaluated between September 2022 and September 2024. IMDs were confirmed by biochemical and/or molecular genetic analyses. Population and live-birth data from the defined referral region were used to estimate regional and NBS-based birth prevalence.

**Results:**

Among 9603 patients evaluated, 828 (8.6%) had a confirmed IMDs; 580 (70.0%) were identified through NBS, 230 (27.8%) through symptomatic referral, and 18 (2.2%) through family screening. Vitamin and cofactor metabolism (43.8%) and amino acid metabolism disorders (35.7%) predominated. Biotinidase deficiency was the most frequent disorder (*n* = 360, 43.4%), including 316 (87.8%) partial and 44 (12.2%) profound cases. Neurological manifestations were the most frequent presentation among symptomatic patients (23.2%). Based on 61,409 estimated live births, estimated birth prevalence was 1:627 for the hyperphenylalaninemia/phenylketonuria spectrum and 1:543 for biotinidase deficiency. Minimum estimated regional prevalence was 1.07 and 0.97 per 100,000 for mucopolysaccharidoses and alkaptonuria, respectively.

**Conclusion:**

NBS and symptom-based evaluation are complementary diagnostic pathways for IMDs. The observed disease spectrum reflects the influence of screening strategies, while symptomatic referrals reveal broader clinical and genetic heterogeneity.

## Highlights

• Newborn screening identified 70% of confirmed inherited metabolic disorders.
• Biotinidase deficiency was the most frequent IMDs, accounting for 43.4%.
• Symptomatic referrals revealed a broad and heterogeneous spectrum of IMDs.
• Regional estimates quantified the minimum recognized burden of selected IMDs.

## 1 Introduction

In recent years, advances in metabolic and genomic diagnostic methods have substantially improved the detection of inborn errors of metabolism. Although the true prevalence of inherited metabolic disorders (IMDs) is thought to remain relatively stable, the number of diagnosed patients has increased owing to improved biochemical and genetic diagnostic approaches. According to the most recent International Classification of Inherited Metabolic Disorders (ICIMD), more than 1500 inherited metabolic disorders have been identified, a substantial proportion of which are considered treatable diseases [1]. The number of recognized IMDs continues to expand with ongoing advances in metabolic and genomic medicine.

IMDs can be challenging to diagnose in conventional clinical settings because of their rarity and clinical variability. Neurologic manifestations such as developmental delay, hypotonia, intellectual disability, and epilepsy are among the most common presentations of IMDs. However, IMDs may affect multiple organ systems and can present from the neonatal period to adulthood with highly heterogeneous clinical findings. Hepatic dysfunction, hypoglycemia, cardiomyopathy, nephrolithiasis, splenomegaly, cataract, and psychiatric manifestations may be additional clinical manifestations in affected individuals [2].

Most IMDs are inherited as monogenic disorders, frequently with autosomal recessive inheritance patterns. Therefore, IMDs are encountered more frequently in populations with relatively high rates of consanguineous marriages. The overall frequency of inherited metabolic disorders has been estimated at approximately 1 in 5000 [3], [4]. The global birth prevalence of IMDs has been estimated at 50.9 per 100,000 live births, compared with 75.7 per 100,000 live births in Middle Eastern populations. IMDs are estimated to account for 23,529 deaths annually in low- and middle-income countries, representing 0.4% of all child deaths [5].

Disorders of amino acid metabolism are among the most frequently reported groups of inherited metabolic disorders, although their relative frequency varies according to population characteristics, diagnostic strategies, and newborn screening practices [5], [6]. In Türkiye, the National Newborn Screening (NBS) program currently includes phenylketonuria (PKU), congenital hypothyroidism, biotinidase deficiency, cystic fibrosis, congenital adrenal hyperplasia, and spinal muscular atrophy [7]. Previous Turkish newborn screening studies have reported frequencies ranging from 1:2407 to 1:7878 for PKU and from 1:481 to 1:2359 for biotinidase deficiency [8], [9].

In this study, Eastern Türkiye was defined as the primary referral catchment area of our center, comprising Erzurum and six surrounding provinces. This region has demographic characteristics that may influence the frequency of autosomal recessive disorders, including relatively high rates of consanguineous marriages compared with some other regions of the country [10]. We aimed to evaluate the diagnostic spectrum and clinical presentation patterns of inherited metabolic disorders identified through newborn screening and symptomatic referrals at a tertiary referral center in Eastern Türkiye.

## 2 Materials and methods

### 2.1 Study design, setting, and population

This retrospective study was conducted at the Inherited Metabolic Diseases Department of a tertiary referral hospital in Eastern Türkiye between September 2022 and September 2024. Patients evaluated through the national NBS program and those referred from other departments because of clinical suspicion of IMDs were included. As the study population consisted of patients referred to a specialized inherited metabolic diseases clinic, it represented a highly selected, high-risk population.

The primary objective was to characterize the spectrum, distribution, and diagnostic pathways of confirmed IMDs within the primary referral region. Accordingly, detailed clinical and diagnostic data were collected for patients with confirmed IMDs. Individuals in whom an IMD was not confirmed were excluded from the disease-spectrum analysis, and detailed phenotypic and final diagnostic data were not systematically collected for this group. Medical records of all patients evaluated in the department during the study period were retrospectively reviewed. After repeated outpatient visits and hospital admissions for the same patient were excluded, the study population comprised 9603 unique patients. Only patients with IMDs confirmed by biochemical and/or molecular genetic analyses were included in the final study cohort.

### 2.2 Referral region and epidemiological estimates

In this study, Eastern Türkiye refers to the primary referral region of our center, comprising Erzurum and six surrounding provinces, with a mean population of approximately 2.16 million during 2022–2024. Province-level live-birth data for the referral region were obtained from the Turkish Statistical Institute (TurkStat) Birth Statistics [11]. As the study covered the period from September 2022 through September 2024, annual live-birth counts were weighted according to the corresponding months of each calendar year, yielding an estimated denominator of 61,409 live births for the study period.

The study was primarily designed to characterize the diagnostic spectrum and experience of a tertiary referral center rather than to determine true population-based prevalence. Therefore, regional epidemiological estimates were interpreted as minimum estimated regional prevalence rates.

Minimum estimated regional prevalence was calculated by dividing the number of confirmed cases originating from the defined referral region by the mean regional population during 2022–2024 and was expressed per 100,000 population. For NBS-related disorders, estimated birth prevalence was calculated using the number of newly diagnosed cases during the study period divided by the estimated number of live births in the same referral region and period.

### 2.3 Newborn screening pathway

During the study period, the Turkish National Newborn Screening Program included six disorders: PKU, congenital hypothyroidism, biotinidase deficiency, cystic fibrosis, congenital adrenal hyperplasia, and spinal muscular atrophy [7]. However, the present study was conducted specifically in the Inherited Metabolic Diseases Department and therefore did not encompass all screen-positive infants identified through the national program. According to institutional referral pathways, infants with suspected hyperphenylalaninemia/PKU and biotinidase deficiency were primarily referred to our department, whereas infants screening positive for congenital hypothyroidism, congenital adrenal hyperplasia, cysticfibrosis,or spinal muscular atrophy were generally referred to the corresponding pediatric subspecialty departments. Consequently, the NBS-derived cohort predominantly comprised patients referred for hyperphenylalaninemia /PKU and biotinidase deficiency.

Patients referred through the NBS program were evaluated according to national screening protocols. In patients with low biotinidase enzyme activity, the diagnosis was confirmed by biochemical and/or molecular genetic analyses. In patients with persistent hyperphenylalaninemia, pterin and dihydropteridine reductase analyses were performed when clinically indicated to exclude tetrahydrobiopterin metabolism disorders, and molecular genetic analyses were used for diagnostic confirmation.

### 2.4 Diagnostic evaluation of symptomatic patients

Symptomatic patients referred from pediatric neurology, gastroenterology, endocrinology, nephrology, hematology, intensive care, and other departments underwent stepwise metabolic evaluation according to their clinical presentation and suspected diagnostic category. Initial laboratory investigations included ammonia, lactate, total homocysteine, and biotinidase enzyme activity, as clinically indicated. Advanced biochemical investigations included plasma amino acid analysis, urine organic acid analysis, acylcarnitine profiling, urine sugar chromatography, and very-long-chain fatty acid analysis according to clinical findings and differential diagnosis.

Patients with clinical findings suggestive of lysosomal storage disorders underwent leukocyte enzyme analyses in referral laboratories, followed by molecular genetic confirmation when enzyme deficiencies were detected. Galactosemia was diagnosed based on reduced galactose-1-phosphate uridyltransferase activity together with elevated galactose metabolites. Additional specialized investigations, including transferrin isoelectric focusing, plasma and urine creatine analysis, and cholestanol measurement, were performed in patients with compatible phenotypic findings.

Clinical exome sequencing was performed in patients who remained undiagnosed despite routine metabolic investigations. Genetic findings were interpreted in conjunction with biochemical and clinical data.

### 2.5 Data collection, classification, and statistical analysis

Demographic characteristics, age at diagnosis, sex distribution, presenting symptoms, diagnostic pathways, diagnostic categories, and treatment modalities were extracted from medical records. Disorders were classified according to the ICIMD [12].

Statistical analyses were performed using SPSS version 22.0 (IBM Corp., Armonk, NY, USA). Categorical variables were summarized as absolute frequencies (n) and percentages (%). Continuous variables were presented as mean ± standard deviation or median (minimum–maximum), according to data distribution. Because the primary aim of the study was descriptive characterization of the diagnostic spectrum, inferential comparative statistical analyses were not prioritized.

## 3 Results

**Fig. 1:**
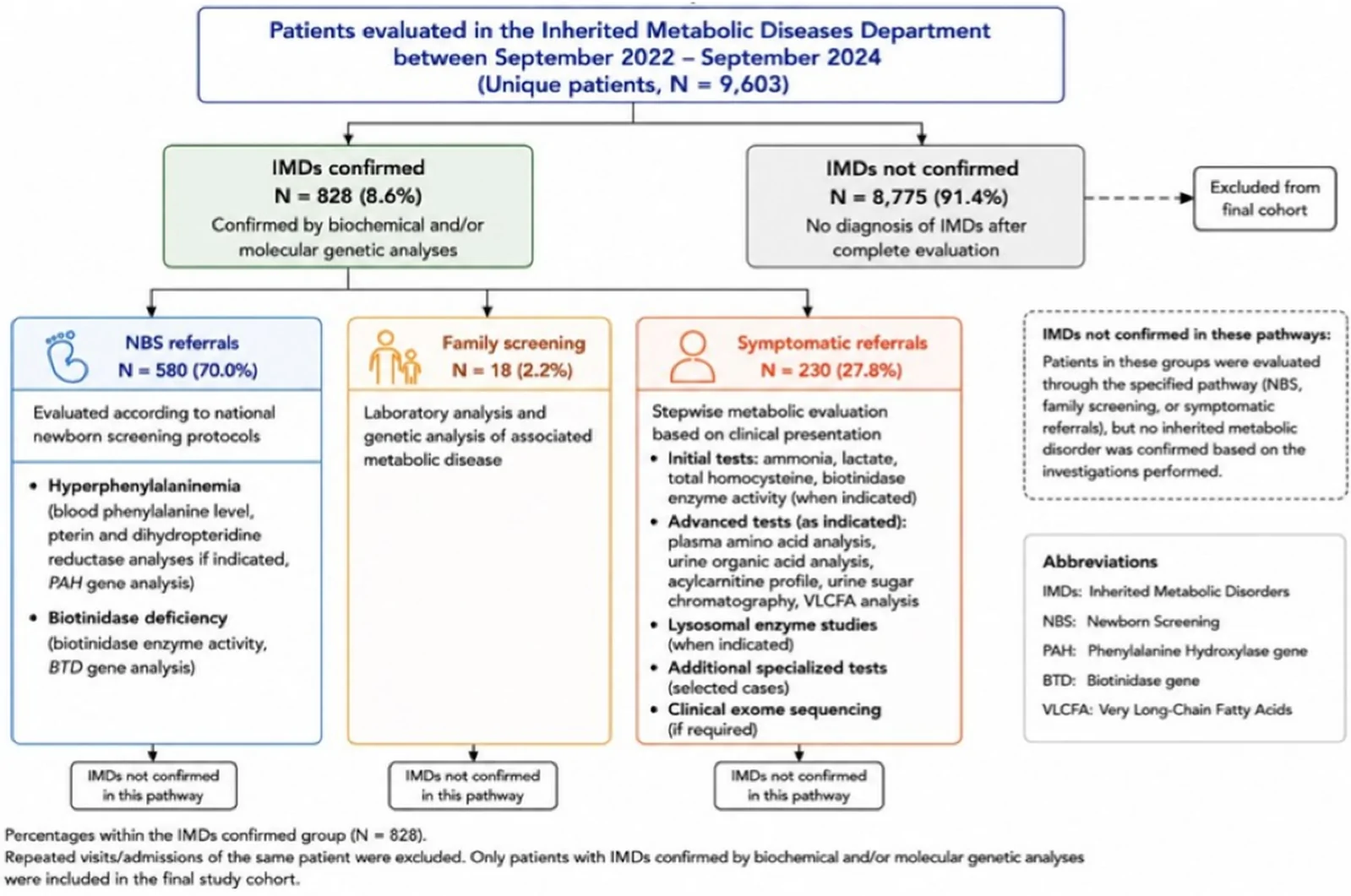
Study flowchart of patients evaluated and diagnosed with inherited metabolic disorders between 2022 and 2024.

**Fig. 2:**
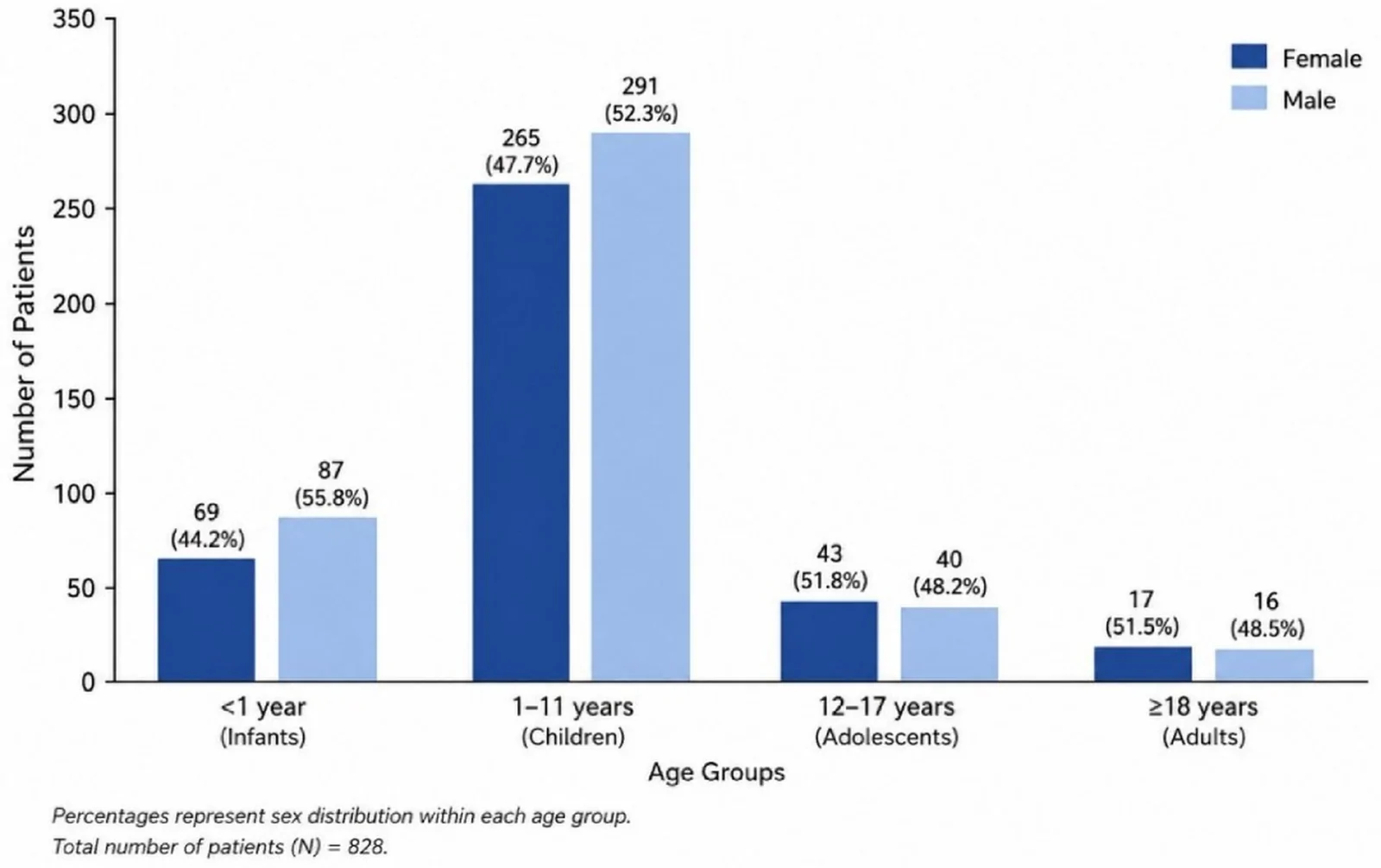
Age group and sex distribution of patients diagnosed with inherited metabolic disorders.

During the study period between September 2022 and September 2024, a total of 9603 unique patients were evaluated in the Department of Inherited Metabolic Diseases. Among these patients, 828 individuals (8.6%, *n* = 828/9603) received a confirmed diagnosis of an IMD based on biochemical and/or molecular investigations (Fig. 1, Table 1). Age at diagnosis ranged from 2 days to 52 years with a male-to-female ratio of 1:1.1 (Fig. 2).

**Table 1:** Clinical diagnoses of patients (between 2022 and 2024).

Category according to ICIMD/Disorders	Number of cases, (%)
Disorders of amino acids metabolism	296 (35.7%)
− Hyperphenylalaninemia	241 (29.1%)
− Alkaptonuria	21 (2.5%)
− Organic acidemia (MSUD, PA, MMA, IVA, 3-MCC deficiency)	15 (1.8%)
− Disorders of sulfur metabolism (classic homocystinuria, MTHFR deficiency, cystathionuria)	6 (<1%)
− Disorders of urea cycle (CPS-1 deficiency, citrullinemia)	4 (<1%)
− Tyrosinemia (type 1/type 2)	3 (<1%)
− Non-ketotic hyperglycinemia	3 (<1%)
− Disorders of amino acid transport (iminoglycinuria, LPI)	2 (<1%)
− Glutaric aciduria type 1	1 (<1%)
Disorders of carbohydrate metabolism	40 (4.8%)
− Galactosemia	15 (1.8%)
− Glycogen storage diseases	15 (1.8%)
− Hereditary fructose intolerance	6 (<1%)
− Fructose 1,6 bisphosphatase deficiency	2 (<1%)
− GLUT-1 deficiency	2 (<1%)
Disorders of fatty acid oxidation	11 (1.3%)
− Glutaric aciduria type 2	6 (<1%)
− Primary carnitine deficiency	2 (<1%)
− Mitochondrial trifunctional protein deficiency	1 (<1%)
− HMG CoA lyase deficiency	1 (<1%)
− HMG CoA synthetase deficiency	1 (<1%)
Disorders of vitamin and co-factor metabolism	363 (43.8%)
− Biotinidase deficiency	360 (43.4%)
− Cerebral folate transporter defect	2 (<1%)
− Dihydropteridine reductase deficiency	1 (<1%)
Disorders of complex molecule degradation	47 (5.6%)
− Mucopolysaccharidoses (MPS 1,3,4 A,6,7)	23 (2.7%)
− Niemann-Pick type B/type C	8 (<1%)
− Mucolipidosis/Oligosaccharidosis	5 (<1%)
− Gaucher type 1	4 (<1%)
− Metachromatic leukodystrophy	2 (<1%)
− Fabry disease	2 (<1%)
− Cystinosis	1 (<1%)
− Tay-Sachs disease	1 (<1%)
− Pompe disease	1 (<1%)
Mitochondrial disorders	17 (2%)
− Krebs cycle disorders	13 (1.5%)
− D2/L2 Hydroxyglutaric aciduria	2 (<1%)
− Pyruvate dehydrogenase deficiency	1 (<1%)
− Ethylmalonic acidemia	1 (<1%)
Disorders of lipid metabolism	7 (<1%)
− Cerebrotendinous xanthomatosis	5 (<1%)
− Peroxisomal disorders	2 (<1%)
Disorders of lipoprotein metabolism	32 (3.8%)
− Familial hypertriglyceridemia	9 (1%)
− Familial hypercholesterolemia	23 (2.7%)
Congenital disorders of glycosylation	5 (<1%)
Neurotransmitter disorders	3 (<1%)
− SSDH deficiency	2 (<1%)
− GABA transaminase deficiency	1 (<1%)
Disorders of energy substrate metabolism	2 (<1%)
− Cerebral creatine transporter deficiency	2 (<1%)
Disorders of peptide and amine metabolism	2 (<1%)
− Glutathione synthetase deficiency	2 (<1%)
Disorders of nucleobase, nucleotide and nucleic acid metabolism	2 (<1%)
− Hereditary xanthinuria type 1	1 (<1%)
− Dihydropyrimidinase deficiency	1 (<1%)
Disorders of trace elements and metals	1 (<1%)
− Mitochondrial membrane protein-associated neurodegeneration (MPAN)	1 (<1%)

Abbreviations: CPS-1, carbamoyl phosphate synthetase 1; LPI, lysinuric protein intolerance; IVA, isovaleric acidemia; MMA, methylmalonic acidemia; MSUD, maple syrup urine disease; MTHFR deficiency, methylenetetrahydrofolate reductase deficiency; PA, propionic acidemia; SSDH deficiency, succinic semialdehyde dehydrogenase.

The national NBS program identified approximately 70.0% (*n* = 580/828) of confirmed IMD cases, and the remaining patients (27.8%, *n* = 230/828) were diagnosed through referral with symptoms from pediatric and adult subspecialty clinics. Family screening led to the diagnosis of 18 patients (2.2%, *n* = 18/828) (Fig. 1).

**Fig. 3:**
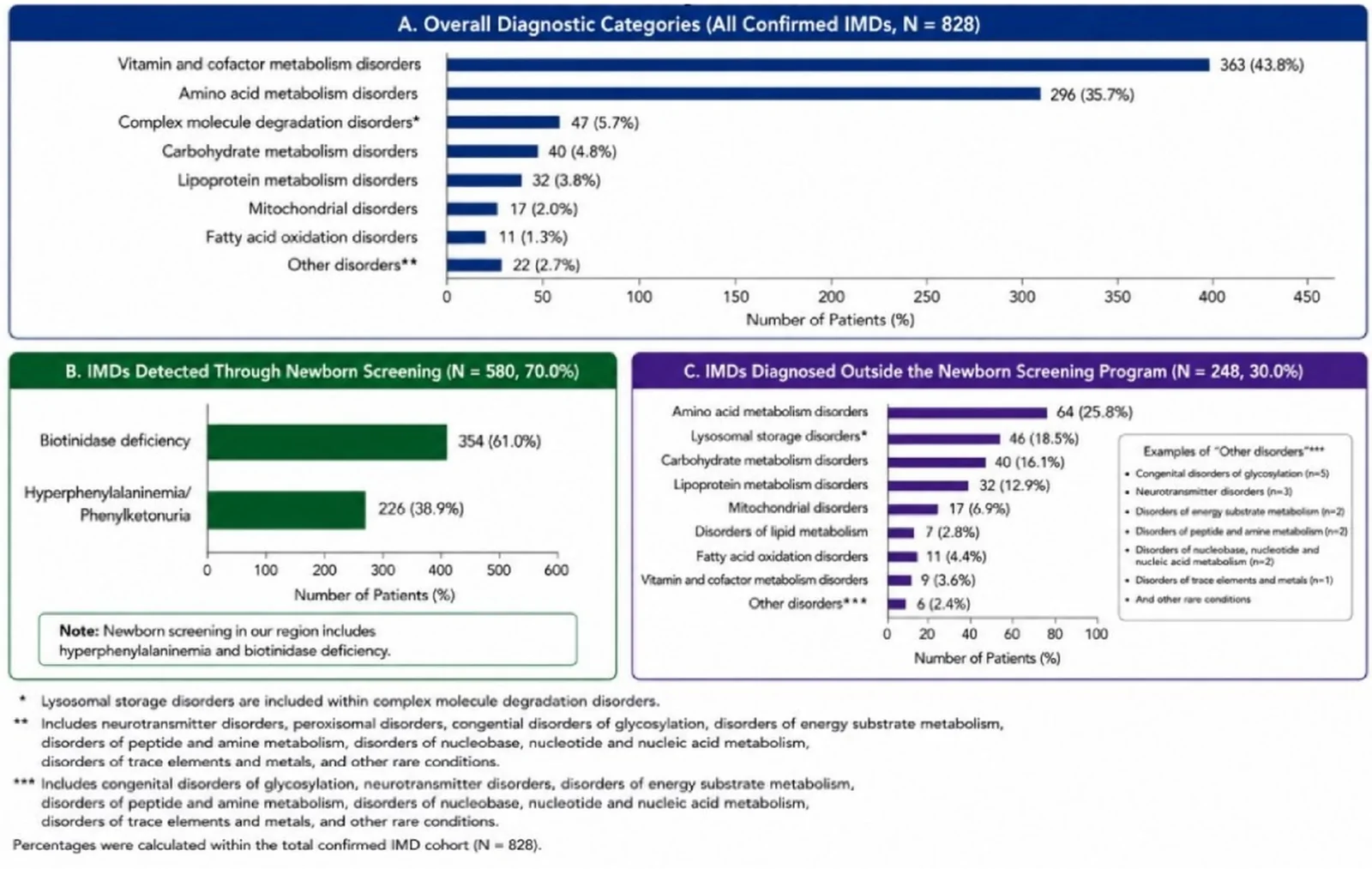
Distribution of inherited metabolic disorders according to diagnostic categories and diagnostic pathways. (A) Distribution according to ICIMD categories, (B) Disorders detected through newborn screening, (C) Disorders diagnosed outside the newborn screening program.

According to the ICIMD classification, vitamin and cofactor metabolism disorders constituted the largest diagnostic category (*n* = 363, 43.8%), followed by amino acid metabolism disorders (*n* = 296, 35.7%) and complex molecule degradation disorders (*n* = 47, 5.6%). The overall distribution of IMDs and the differences in diagnostic spectrum between NBS and non-NBS pathways are illustrated in Fig. 3, while detailed diagnostic categories are presented in Table 1.

Among patients diagnosed through the NBS program, biotinidase deficiency and hyperphenylalaninemia/PKU constituted the majority of cases, reflecting the current national screening panel in Türkiye. Disorders of vitamin and cofactor metabolism represented the largest diagnostic category overall, predominantly due to biotinidase deficiency (99.1%, *n* = 360/363). Of these patients, 316 (87.8%) were classified as having partial biotinidase deficiency and 44 (12.2%) as having profound biotinidase deficiency. Disorders of amino acid metabolism constituted the second largest category (35.7%, *n* = 296/828), primarily consisting of hyperphenylalaninemia/PKU (81.4%, *n* = 241/296) (Table 1). In the hyperphenylalaninemia group, 31.5% (*n* = 76/241) of the patients were classical PKU, 20.3% (*n* = 49/241) tetrahydrobiopterin-responsive PKU, and 48.1% (*n* = 116/241) persistent hyperphenylalaninemia requiring long-term follow-up. Among the organic acidemias, methylmalonic acidemia accounted for 53.3% (*n* = 8/15), 3-methylcrotonyl-CoA carboxylase deficiency for 20.0% (*n* = 3/15).

During the study period, patients were newly diagnosed with hyperphenylalaninemia/phenylketonuria and 113 with biotinidase deficiency through the newborn screening pathway. All of these patients originated from the defined referral region. Based on an estimated 61,409 live births in the same region during the corresponding study period, the estimated birth prevalence was 159.6 per 100,000 live births (approximately 1:627) for hyperphenylalaninemia/PKU and 184 per 100,000 live births (approximately 1:543) for biotinidase deficiency.

Using the mean population of the referral region during 2022–2024 (approximately 2.16 million) as the denominator, minimum estimated regional prevalence rates were calculated for selected IMDs (Table 2). The highest estimates were observed for biotinidase deficiency (16.70 per 100,000; approximately 1:5988) and hyperphenylalaninemia/PKU (11.18 per 100,000; approximately1:8944). Among disorders not primarily identified through the national newborn screening pathway, mucopolysaccharidoses (MPS) and familial hypercholesterolemia each had a minimum estimated prevalence of 1.07 per 100,000 (approximately 1:93,718), followed by alkaptonuria at 0.97 per 100,000 (approximately 1:102,644). Minimum estimated regional prevalence rates for other selected IMDs are presented in Table 2.

**Table 2:** Minimum estimated regional prevalence of selected inherited metabolic disorders diagnosed in the referral region.

Disorder	Confirmed cases, n	Minimum estimated regional prevalence per 100,000	Approximate frequency
Biotinidase deficiency	360	16.70	1:5988
Hyperphenylalaninemia/PKU	241	11.18	1:8944
Mucopolysaccharidoses	23	1.07	1:93,718
Familial hypercholesterolemia	23	1.07	1:93,718
Alkaptonuria	21	0.97	1:102,644
Galactosemia	15	0.70	1:143,701
Glycogen storage diseases	15	0.70	1:143,701
Krebs cycle disorders	13	0.60	1:165,809
Familial hypertriglyceridemia	9	0.42	1:239,502
Hereditary fructose intolerance	6	0.28	1:359,253
Glutaric aciduria type 2	6	0.28	1:359,253
Cerebrotendinous xanthomatosis	5	0.23	1:431,103

Minimum estimated regional prevalence was calculated using the mean population of the primary referral region during 2022–2024 (approximately 2.16 million) as the denominator. All included cases originated from the defined referral region. These estimates represent diagnosed cases identified and followed at a tertiary referral center and should therefore be interpreted as minimum regional prevalence estimates rather than true population-based prevalence.

Symptomatic referrals demonstrated substantial phenotypic heterogeneity and multisystem involvement. Detailed data on presenting manifestations were available for 202 symptomatic patients and were included in the clinical presentation analysis. The most common reason for metabolic evaluation was neurological manifestations, accounting for 23.2% (*n* = 47/202) of symptomatic presentations. Developmental delay/intellectual disability was the most frequent neurological finding (76.6%, *n* = 36/47), followed by seizures (8.5%, *n* = 4/47) and hypotonia (8.5%, *n* = 4/47). Additional presenting features included isolated transaminase elevation (6.8%, *n* = 14/202), hypoglycemia (5.4%, *n* = 11/202), hepatosplenomegaly (5.4%, *n* = 11/202), cataract (4.9%, *n* = 10/202), dysostosis multiplex, coarse facial features, and dark urine discoloration (7.9%, *n* = 16/202).

Disorders of complex molecule degradation represented the third most frequent diagnostic category (5.5%, *n* = 46/828). MPS accounted for 50% (*n* = 23/46) of this group, with MPS type VI representing 60.8% (*n* = 14/23) of all MPS cases. Niemann-Pick disease was responsible for 17% (*n* = 8/47) of complex molecule degradation disorders.

The study also identified several rare but potentially treatable neurometabolic disorders, including cerebral creatine transporter deficiency (*n* = 2), cerebral folate transporter deficiency (*n* = 2), cerebrotendinous xanthomatosis (*n* = 5), and neurotransmitter disorders (*n* = 3) (Table 1). These diagnoses were primarily made through targeted metabolic investigations and clinical exome sequencing in patients with unexplained neurodevelopmental findings.

Treatment approaches varied across disease categories. Most patients (53.0%, *n* = 355/670) received biotin supplementation, followed by tetrahydrobiopterin (7.3%, *n* = 49/670). Disease-specific dietary treatment was most common for classical PKU (46.5%, *n* = 63/135), galactosemia (9.3%, *n* = 13/135), organic acidemias (8.6%, *n* = 12/135), glycogen storage diseases (6.2%, *n* = 8/135), and hereditary fructose intolerance (3.7%, *n* = 5/135). Twenty-three patients received enzyme replacement therapy, most commonly for MPS type VI (47.8%,*n* = 11/23),Gaucher disease type I (17.3%,*n* = 4/23), and MPS type IV A(13.0%,*n* = 3/23).

Overall, the findings demonstrate that newborn screening programs are highly effective for identifying specific treatable disorders included in the national screening panel, whereas symptomatic referral pathways remain essential for the diagnosis of clinically heterogeneous multisystem inherited metabolic diseases not detectable through routine newborn screening.

## 4 Discussion

This study provides a comprehensive overview of the spectrum of IMDs diagnosed at a major tertiary referral center serving approximately 2.16 million people in Eastern Türkiye and highlights the complementary roles of NBS and symptom-based metabolic evaluation. Three principal findings emerged from our study. First, disorders of vitamin and cofactor metabolism constituted the largest diagnostic category, predominantly because of the high number of patients with biotinidase deficiency identified through NBS. Second, although NBS accounted for approximately 70% of confirmed diagnoses, symptomatic referrals revealed a substantially broader and clinically heterogeneous spectrum of IMDs (Fig. 3). Third, the distribution of IMDs in our cohort differed from several internationally reported cohorts, emphasizing the influence of population characteristics, referral pathways, diagnostic strategies, and the composition of national newborn screening programs on the spectrum of disorders identified in tertiary metabolic centers.

Recent advances in biochemical diagnostics and next-generation sequencing have considerably expanded the recognized spectrum of IMDs. The International Classification of Inherited Metabolic Disorders currently encompasses more than 1500 disorders, and this number continues to increase with ongoing genomic discoveries [1], [12].In our cohort, clinical exome sequencing contributed particularly to the diagnosis of patients with atypical or unexplained neurodevelopmental phenotypes, including rare and potentially treatable disorders such as cerebral creatine transporter deficiency, cerebral folate transporter deficiency, cerebrotendinous xanthomatosis, and neurotransmitter disorders. These findings support the increasing integration of genomic testing with biochemical and phenotypic evaluation in contemporary metabolic diagnostic pathways.

Marked differences were observed when our diagnostic distribution was compared with recently published international cohorts. In our study, vitamin and cofactor metabolism disorders constituted 43.8% of all confirmed IMDs, followed by amino acid metabolism disorders (35.7%), complex molecule degradation disorders (5.6%), carbohydrate metabolism disorders (4.8%), and lipoprotein metabolism disorders (3.8%). In contrast, Georgiou et al. evaluated 7388 patients over 33 years in Cyprus and confirmed an IMD in 200 patients, corresponding to a diagnostic yield of 2.7%. Amino acid metabolism disorders constituted 41.0% of their cohort, followed by carbohydrate metabolism disorders (16.5%), complex molecule degradation disorders (16.5%), mitochondrial disorders (10.5%), and vitamin and cofactor metabolism disorders (5.5%). Hyperphenylalaninemia was their most frequent individual diagnosis (14.0%), followed by galactosemia (7.0%), glutaric aciduria type I (5.5%), and maple syrup urine disease (4.0%) [6]. Thus, although amino acid disorders were common in both populations (35.7% vs. 41.0%), vitamin and cofactor disorders were almost eightfold more prominent in our cohort (43.8% vs. 5.5%), largely because of the high contribution of biotinidase deficiency to our NBS-derived population.

A large tertiary-center study from India also demonstrated a markedly different disease distribution. Sheth et al. reported 3294 patients with 305 rare genetic disorders identified over 22 years; 1992 patients (60.5% of all diagnosed patients) had an IMD, encompassing 47 distinct metabolic diseases. Gaucher disease was the most frequent IMDs, accounting for 224 cases (11.2% of the IMDs group) [13]. In comparison, Gaucher disease was uncommon in our cohort (*n* = 4), whereas biotinidase deficiency (*n* = 360, 43.4%) and contemporary metabolic hyperphenylalaninemia/PKU (*n* = 241, 29.1%) together accounted for 72.5% of all confirmed IMDs. The Indian cohort also demonstrated the importance of population-specific genetic architecture, with founder variants identified for disorders including Tay–Sachs disease and MPS IVA [13]. These substantial differences illustrate how referral structure, population genetics, and screening practices can markedly alter the apparent distribution of IMDs across tertiary-center cohorts.

The influence of NBS strategy becomes even more apparent when our findings are compared with expanded screening programs. In Iran, Shakiba et al. screened 125,819 newborns for 46 IMDs using tandem mass spectrometry and identified 124 confirmed cases, corresponding to an overall prevalence of approximately 1:1015. Aminoacidopathies were the most frequently detected group, and hyperphenylalaninemia/PKU was the most common individual disorder [14]. These marked geographic differences illustrate the combined effects of population genetics, screening-panel composition, and disease definitions on reported IMDs frequencies.

In contrast to these expanded NBS programs, the metabolic disorders represented through the Turkish NBS referral pathway in our center during the study period were predominantly hyperphenylalaninemia/PKU and biotinidase deficiency. Consequently, 354 of the 580 NBS-derived confirmed diagnoses (61.0%) were biotinidase deficiency and 226 (39.0%) were hyperphenylalaninemia/PKU. This differs substantially from expanded MS/MS-based programs, in which organic acidemias and fatty acid oxidation disorders constitute additional major diagnostic groups [15], [16], [17]. For example, the Iranian expanded NBS study ranked aminoacidopathies first, followed by organic acidemias and fatty acid oxidation disorders [14]. In our entire cohort, by contrast, fatty acid oxidation disorders accounted for only 11 of 828 diagnoses (1.3%). These findings indicate that differences in screening-panel composition contribute substantially to the contrasting diagnostic spectra reported across countries.

The birth-prevalence analysis further illustrates the regional contribution of the two principal disorders identified through NBS. During the study period, 98 patients were newly diagnosed with hyperphenylalaninemia/PKU and 113 with biotinidase deficiency among an estimated 61,409 live births in the referral region, corresponding to estimated birth prevalences of 159.6 per 100,000 (approximately 1:627) and 184.0 per 100,000 (approximately 1:543), respectively. Previous Turkish studies have similarly demonstrated relatively high frequencies of both disorders [8], [9]. However, the hyperphenylalaninemia/PKU estimate in the present study should not be interpreted as the prevalence of classical PKU alone. Among all 241patients in the hyperphenylalaninemia/PKU group, only 76 (31.5%) had classical PKU, whereas 49 (20.3%) had tetrahydrobiopterin-responsive PKU and 116 (48.1%) had persistent hyperphenylalaninemia. Differences in diagnostic definitions are therefore important when comparing hyperphenylalaninemia/PKU frequencies between studies.

Biotinidase deficiency was particularly prominent in our cohort, accounting for 360 of 828 confirmed IMDs (43.4%). Of these, 316 (87.8%) had partial and 44 (12.2%) had profound biotinidase deficiency. This distribution provides important context for the high contribution of vitamin and cofactor metabolism disorders to our overall cohort. The estimated birth prevalence of biotinidase deficiency in our referral region was approximately 1:543, broadly supporting previous reports indicating a relatively high frequency of biotinidase deficiency in Türkiye [8], [9]. The predominance of partial deficiency should also be considered when comparing our overall biotinidase frequency with studies using different biochemical thresholds or reporting only profound deficiency.

The regional prevalence analysis provides an additional perspective beyond the NBS- derived findings. Using a mean referral-region population of approximately 2.16 million, the minimum estimated regional prevalence was 1.07 per 100,000 for mucopolysaccaridoses, 0.97 per 100,000 for alkaptonuria, 0.70 per 100,000 for galactosemia and glycogen storage diseases, 0.60 per 100,000 for krebs cycle disorders, and 0.23 per 100,000 for cerebrotendinous xanthomatosis. These estimates should not be interpreted as true population-based prevalence because ascertainment depended on diagnosis and referral to our tertiary center. Nevertheless, they provide an estimate of the minimum recognized disease burden within a geographically defined referral population.

Symptomatic referrals constituted 230 of 828 confirmed cases (27.8%) and revealed a substantially more heterogeneous disease spectrum than the NBS-derived cohort. Detailed presenting manifestations were available for 202 patients. Neurological manifestations were the most common indication for metabolic evaluation, accounting for 47 of 202 patients (23.2%); among these, developmental delay/intellectual disability was present in 36 (76.6%), while seizures and hypotonia were each observed in four patients (8.5%). Neurological involvement is widely recognized as a major presentation of IMDs because of the high metabolic demands and vulnerability of the central nervous system [2], [18]. Several of the neurometabolic conditions identified in our cohort are potentially treatable, reinforcing the importance of considering metabolic etiologies in patients with unexplained neurodevelopmental phenotypes [19], [20], [21], [22].

Multisystem manifestations also provided important diagnostic clues. Isolated transaminase elevation was present in 14 of 202 symptomatic patients (6.9%), hypoglycemia in 11 (5.4%), hepatosplenomegaly in 11 (5.4%), and cataract in 10 (5.0%). Hepatic abnormalities were particularly associated with glycogen storage diseases, galactosemia, mitochondrial disorders, and hereditary fructose intolerance, whereas cataract, corneal abnormalities, dysostosis multiplex, coarse facial features, hepatosplenomegaly, and dark urine discoloration facilitated recognition of lysosomal storage disorders and alkaptonuria [23], [24], [25], [26]. These findings illustrate why symptom-based metabolic evaluation remains indispensable even in healthcare systems with established NBS programs.

The study has several limitations. First, although our center is a major tertiary referral center for the defined region, this was not a population-wide active case-ascertainment study; consequently, the calculated regional prevalence figures should be interpreted as minimum estimates rather than true population-based prevalence. Second, the diagnostic distribution was strongly influenced by the structure of the national NBS program and institutional referral pathways, resulting in overrepresentation of biotinidase deficiency and hyperphenylalaninemia/PKU. Third, detailed referral indications, presenting manifestations, and subsequent diagnoses were not systematically available for the 8775 individuals in whom an IMDs was not confirmed, precluding direct comparison between confirmed and unconfirmed referrals. Finally, patients with mild, atypical, or as-yet undiagnosed IMDs may not have been captured. Nevertheless, the inclusion of 828 confirmed patients from a defined referral region, together with complementary NBS and symptomatic diagnostic pathways, live-birth denominators, and regional prevalence estimates, provides a broad real-world representation of the IMDs spectrum encountered in Eastern Türkiye.

Overall, our findings demonstrate that NBS and symptom-based metabolic evaluation are complementary diagnostic strategies. While NBS enables early identification of selected prevalent and treatable disorders, a substantial and clinically heterogeneous group of IMDs continues to depend on clinical recognition, specialized biochemical testing, and genomic investigation. Differences between our cohort and international reports further emphasize that the observed spectrum of IMDs is strongly influenced by screening strategies, referral pathways, and population characteristics.

## Declaration of generative AI in scientific writing

ChatGPT (OpenAI, San Francisco, CA) was used for English language editing. The authors are responsible for the scientific content of this manuscript.

## CRediT authorship contribution statement

**Sezai Arslan:** Writing – original draft, Validation, Supervision, Resources, Project administration, Methodology, Investigation, Funding acquisition, Formal analysis, Data curation, Conceptualization. **Filiz Ekinci Uğan:** Data curation, Conceptualization. **Oğuzhan Yaralı:** Investigation, Data curation, Conceptualization. **Hasan Kahveci:** Supervision, Methodology, Data curation, Conceptualization.

## Informed consent

Requirement for informed consent was waived due to retrospective design.

## Ethical approval

It was obtained from the Erzurum Medical Faculty Scientific Research Ethics Committee (decision no: 2024/09–173).

## Ethics declaration

Verbal informed consent to take part in the study and to publish the article was obtained from all participants or their legal representatives. This retrospective study's purpose, rationale, approach, and methodology have been reviewed, and it has been determined that there are no ethical concerns according to the Scientific Research Ethics Committee guidelines. The privacy rights of participants have been observed.

This study was performed in compliance with relevant laws, regulatory frameworks and guidelines where the research took place.

This study was approved by the Erzurum Medical Faculty Scientific Research Ethics Committee.

(Approval No. 2024/09–173)

## Funding

No external funding was received for this study.

## Declaration of competing interest

The authors have no conflicts of interest to declare. Funding: The authors declared that this study has received no financial support.

## Data Availability

The data that support the findings of this study are available on request from the corresponding author.

## References

[1] den Hollander B., Hoytema van Konijnenburg E.M.M., Hewitson B. (2025). The metabolic treatabolome and inborn errors of metabolism knowledgebase therapy tool: do not miss the opportunity to treat!. J. Inherit. Metab. Dis..

[2] Saudubray J.M., García-Cazorla Á., Saudubray J.M., Baumgartner M.R., García-Cazorla Á., Walter J.H. (2022). Inborn Metabolic Diseases: Diagnosis and Treatment.

[3] Seymour C.A., Thomason M.J., Chalmers R.A., Addison G.M., Bain M.D., Cockburn F. (1997). Newborn screening for inborn errors of metabolism: a systematic review. Health Technol. Assess..

[4] Sanderson S., Green A., Preece M.A., Burton H. (2006). The incidence of inherited metabolic disorders in the West Midlands, UK. Arch. Dis. Child..

[5] Waters D., Adeloye D., Woolham D., Wastnedge E., Patel S., Rudan I. (2018). Global birth prevalence and mortality from inborn errors of metabolism: a systematic analysis of the evidence. J. Glob. Health.

[6] Georgiou T., Petrou P.P., Malekkou A. (2024). Inherited metabolic disorders in Cyprus. Mol. Genet. Metab. Rep..

[7] Republic of Türkiye Ministry of Health (2026). General Directorate of Public Health. Newborn Screening Program (NTP). https://hsgm.saglik.gov.tr/tr/tarama-programlari/ntp.html.

[8] Toktaş İ., Sarıbaş S., Canpolat S., Erdem Ö., Özbek M.N. (2022). Evaluation of patients diagnosed with phenylketonuria and biotinidase deficiency by the newborn screening program: a ten-year retrospective study. Turk. J. Pediatr..

[9] Küçükkelepçe O., Konyalıoğlu F.S., Kurt O. (2024). Incidence analysis of six diseases in the national newborn screening program: a retrospective study from Adıyaman, Turkiye (2019–2023). Anatolian Curr. Med. J..

[10] Turkish Statistical Institute (TurkStat) (2025). https://veriportali.tuik.gov.tr/en/press/53898.

[11] Turkish Statistical Institute (TurkStat) (2025). https://veriportali.tuik.gov.tr/Bulten/Index?dil=1&p=Dogum-Istatistikleri-2022-49673.

[12] Ferreira C.R., Rahman S., Keller M., Zschocke J., ICIMD Advisory Group (2021). An international classification of inherited metabolic disorders (ICIMD). J. Inherit. Metab. Dis..

[13] Sheth J., Nair A., Sheth F. (2024). Burden of rare genetic disorders in India: twenty-two years’ experience of a tertiary Centre. Orphanet J. Rare Dis..

[14] Shakiba M., Yasaei M., Saneifard H. (2024). Expanded inherited metabolic diseases screening by tandem mass spectrophotometry: the first report from Iran. Mol. Genet. Metab. Rep..

[15] Zhang X., Ji W., Wang Y., Zhou Z., Guo J., Tian G. (2024). Comparative analysis of inherited metabolic diseases in normal newborns and high-risk children: insights from a 10-year study in Shanghai. Clin. Chim. Acta.

[16] Couce M.L., Bóveda M.D., Castiñeiras D.E. (2024). A newborn screening programme for inborn errors of metabolism in Galicia:22yearsofevaluationandfollow-up. Orphanet J. Rare Dis..

[17] Ibarra-González I., Fernández-Lainez C., Vela-Amieva M. (2023). A review of disparities and unmet newborn screening needs over 33 years in a cohort of Mexican patients with inborn errors of intermediary metabolism. Int. J. Neonatal Screen..

[18] Hoytema van Konijnenburg E.M.M., Wortmann S.B., Koelewijn M.J., Tseng L.A., Houben R., Stöckler-Ipsiroglu S. (2021). Treatable inherited metabolic disorders causing intellectual disability: 2021 review and digital app. Orphanet J. Rare Dis..

[19] Saudubray J.M., Garcia-Cazorla À. (2018). Inborn errors of metabolism overview: pathophysiology, manifestations, evaluation, and management. Pediatr. Clin. N. Am..

[20] Nashef L., Singh R., Moran N., Murphy E. (2019). Investigating adults with early-onset epilepsy and intellectual or physical disability. Pract. Neurol..

[21] Karadağ M., Turan M.İ., Çelebi C., Caglar T. (2024). Cerebrotendinous xanthomatosis disease prevalence in patients with autism spectrum disorder: a prospective observational study. Mol Syndromol..

[22] İnci A., Özaslan A., Okur İ., Biberoğlu G., Güney E., Ezgü F.S. (2021). Autism: screening of inborn errors of metabolism and unexpected results. Autism Res..

[23] Ferreira C.R., Cassiman D., Blau N. (2019). Clinical and biochemical footprints of inherited metabolic diseases. II. Metabolic liver diseases. Mol. Genet. Metab..

[24] Fenzl C., Teramoto K., Moshirfar M. (2015). Ocular manifestations and management recommendations of lysosomal storage disorders I: mucopolysaccharidoses. Clin. Ophthalmol..

[25] Lanzl I., Leroy B.P. (2012). Ocular features of treatable lysosomal storage disorders—Fabry disease, mucopolysaccharidoses I, II and VI and Gaucher disease. Touch Rev. Ophthalmol..

[26] Cieszyński K., Podgórny J., Mostowska A., Jagodziński P.P., Grzegorzewska A.E. (2016). Alkaptonuria: a disease with dark brown urine. Pol. Arch. Intern. Med..

